# Factors influencing C-reactive protein status on admission in neonates after birth

**DOI:** 10.1186/s12887-024-04583-8

**Published:** 2024-02-01

**Authors:** Chuanding Cao, Shuo Wang, Yang Liu, Shaojie Yue, Mingjie Wang, Xiaohe Yu, Ying Ding, Mei Lv, Keren Fang, Meiyan Chu, Zhengchang Liao

**Affiliations:** Department of Neonatology, Xiangya Hospital, Central South University, No. 87 Xiangya Road, Changsha, Hunan 410008 China

**Keywords:** Neonates, C reactive protein, Perinatal factors, Gestational age, Smooth curve fitting

## Abstract

**Objective:**

To explore the factors influencing C-reactive protein (CRP) status in neonates on admission after birth.

**Methods:**

820 newborns born and hospitalized at Xiangya Hospital of Central South University from Jan. 2020 to Dec. 2020 were retrospectively analyzed. Maternal medical history and medication use during pregnancy, neonatal demographic information and status at birth were collected through the electronic medical record system. Statistical software was used to analyze the possible relationship between perinatal factors and CRP on admission after birth.

**Results:**

A total of 820 neonates were analyzed, including 463 males and 357 females with a mean gestational age (GA) of 36.07 ± 3.30 weeks. (1) Multifactor Logistic regression analysis: larger GA (OR: 1.13, 95%CI: 1.00-1.28, *P* = 0.042), premature rupture of membranes (PROM) ≥ 18 h (OR: 2.39, 95%CI: 1.35–4.23, *P* = 0.003) and maternal autoimmune diseases (OR: 5.30, 95%CI: 2.15–13.07, *P* < 0.001) were independent risk factors for CRP ≥ 8 mg/L. Cesarean delivery (OR 0.40, 95%CI: 0.26–0.60, *P* < 0.001) was independent protective factor for CRP ≥ 8 mg/L. (2) Threshold effect analysis: A non-linear relationship was found between GA and CRP. When GA is less than 33.9 weeks, the risk of CRP ≥ 8 mg/L was reduced by 28% with one week increased (*P* < 0.001), and when GA is more than 33.9 weeks, the risk of CRP ≥ 8 mg/L was increased by 61% with one week increased (*P* < 0.001).

**Conclusions:**

GA, PROM, maternal autoimmune diseases and cesarean delivery were all independent influences neonatal CRP ≥ 8 mg/L on admission, and there was a nonlinear relationship between GA and neonatal CRP ≥ 8 mg/L on admission.

## Introduction

The role of C-reactive protein (CRP) in the inflammatory response has been confirmed, and its level is closely related to the health status of the human body [1]. It is significantly increased in emergencies such as bacterial infections, inflammation, tissue damage, malignant tumors [1]. Serum CRP is well recognized as one of the major biomarkers for the diagnosis of early onset neonatal sepsis (EOS) [2].

Neonatal sepsis is the third major cause of neonatal death and disability in the first month after birth [3]. The incidence of neonatal sepsis varies by different country and income. In the United States and China, the incidence of EOS is approximately 0.8–1.1/1000 live births and 22/1000 live births, respectively [4, 5]. In economically underdeveloped regions, EOS can account for an estimated 30 to 50% of all neonatal deaths each year [6].. Blood culture is the gold standard for the diagnosis of neonatal sepsis, but its long testing period and atypical symptoms in early neonates make early diagnosis of EOS difficult [7]. Therefore, CRP, procalcitonin (PCT) and interleukin-6 (IL-6) are more informative for the early diagnosis of neonatal EOS. High CRP status is indicative of severe bacterial infections and is associated with an increased risk of EOS [2, 8–10]. Salih et al. reported [9] that when CRP is ≥ 8 mg/L, it can be used as one of the reference factors for diagnosing positive blood cultures. Gomathi et al. found [11] that CRP measured within 6 h of the onset of clinical sepsis signs had sensitivity 83%and negative predictive value (NPV) 82.3% in diagnosing neonatal sepsis. After considered the presence of neonatal sepsis, neonates often receive antibiotics for more than 3 days. However, when CRP is used to diagnose neonatal infectious diseases, its specificity is not high [12, 13]. There are multiple other pathological situations, aside from infections, associated with an increase in CRP. Nuntnarumit et al. reported [14] that CRP is indicative for the diagnosis of EOS, leading to prolonged antibiotic use in neonatal intensive care units (NICUs) usually due to high CRP status. This inevitably leads to overuse of antibiotics, resulting in the development and spread of antibiotic resistance [15] and other adverse outcomes such as neonatal necrotizing enterocolitis (NEC) and late-onset sepsis (LOS) [16]. And excessive antibiotic therapy increases the risk of abnormal bacterial colonization, increase in resistant bacteria, and allergic manifestations [16, 17].

Previous descriptive studies on the linear and nonlinear relationships between influencing factors and CRP status are rare, and the lack of quantification of the ability of influencing factors to affect CRP status has led to the inability to estimate the magnitude of the effect, even though it is known that certain factors have an effect on CRP status. This research intends to explore the possible relationship between influencing factors and CRP status, quantify the magnitude of influence of influencing factors on CRP status through regression analysis, and attempt to explore whether there is a nonlinear relationship influence of certain factors, which will help physicians to better assess the risk of infections in neonates, administer antimicrobial drugs in a timely manner, and also avoid overuse of antibiotic drugs.

## Materials and methods

### Research Population

This was retrospective cross-sectional research of 872 neonates born at Xiangya Hospital of Central South University between Jan. 2020 and Dec. 2020 and admitted to the Neonatal Intensive Care Unit within 2 h of birth. The CRP status of all the above neonates were measured on admission. 40 neonates had to be excluded because of incomplete data; 4 neonates had to be excluded because of positive blood culture; 6 neonates had to be excluded because of combined with congenital developmental malformation, 2 neonates had to be excluded because of died within 48 h after admission. All clinical, perinatal, neonatal and laboratory data were cross-checked for accuracy. A final total of 820 neonates were enrolled, and the neonates were categorized into two groups based on whether or not they had a CRP value of ≥ 8 mg/L: the CRP ≥ 8 mg/L group (163 cases, 98 males, 65 females, mean GA 37.71 ± 3.51 weeks) and the CRP < 8 mg/L group (657 cases, 365 males, 292 females, mean GA 35.66 ± 3.11 weeks).

### Determination of CRP

CRP status were measured by immunoturbidimetric assay (CRP kit, Beckman Coulter, USA) and IM-MAGE800 fully automated special protein analysis system (Beckman Coulter, USA). The cut-off values of CRP were 8 mg/L [18, 19].

### Ethics Statement

The studies involving human participants were reviewed and approved by the Ethics Committee of Xiangya Hospital, Central South University and has been carried out in accordance with the Declaration of Helsinki (2000) of the World Medical Association. Written informed consent to participate in this research was provided by the participants’ legal guardian/next of kin. (Ethics Review Department No. 202307164).

### Statistical analysis

If the continuous variable was normally distributed, it was expressed as mean ± standard deviation (SD). Categorical variables were expressed in frequency or as a percentage. χ ^2^ (categorical variables), Student’s t-test (normal distribution), or Mann–Whitney U-test (skewed distribution) were utilized to analyze differences between CRP ≥ 8 mg/L group and CRP<8 mg/L group.

Univariate analysis and multiple logistic regression were used to analyze the possible association between gestation age, PROM, antenatal steroids, maternal autoimmune diseases, delivery mode, MAS and the incidence of CRP ≥ 8 mg/L. Two models were constructed to illustrate the stability of this relationship: Model 1 adjust for: sex, gestational age, birth weight; Model 2 adjust for: sex, GA, birth weight (BW), maternal fever, placenta previa, antenatal antibiotic use, PROM, pregnancy hypertension, gestational diabetes, ICP, MAS, antenatal steroids, maternal autoimmune diseases. To address non-linearity of gestation age and the incidence of CRP ≥ 8 mg/L, a generalized additive model and smooth curve fitting (penalized spline method) were conducted. After non-linearity was detected, we first calculated the inflection point using a recursive algorithm and then constructed a two-piecewise logistic regression on both sides of the inflection point. We determined the best fit model based on the *P*-values for the log likelihood ratio test. All the analyses were performed with the statistical software packages R (version 3.6.1) (http://www.R-project.org, The R Foundation) and EmpowerStats (http://www.empowerstats.com, X&Y Solutions, Inc, Boston, MA). *P*-values < 0.05 (two-sided) were considered statistically significant.

## Results

### Comparison of baseline characteristics

We presented the basic characteristics of the two groups in Table 1. We found statistical differences in gestational age (GA), birth weight (BW), premature rupture of membrane (PROM) (≥ 18 h), antenatal steroids, placenta previa, maternal autoimmune diseases, intrahepatic cholestasis of pregnancy (ICP), delivery mode (Cesarean delivery), and meconium aspiration syndrome (MAS) between the two groups. GA and BW in CRP ≥ 8 mg/L group were significantly larger than that in CRP<8 mg/L group. The incidence rate of CRP ≥ 8 mg/L was significantly higher when neonates exposed to PROM(≥ 18 h), maternal autoimmune diseases and MAS; and significantly lower when newborns exposed to antenatal steroids, placenta previa, ICP and cesarean delivery. (Table 1)

**Table 1:** Basic characteristics of the CRP ≥ 8 mg/L group compared with the CRP<8 group.[Mean ± SD, *n* (%)]

	CRP<8 mg/L(*n* = 657)	CRP ≥ 8 mg/L(*n* = 163)	*P*-value
GA	35.66 ± 3.11	37.71 ± 3.51	0.001
BW	2472.00 ± 766.69	2987.58 ± 873.21	0.001
Maternal fever			0.321
No	648 (98.63%)	159 (97.55%)	
Yes	9 (1.37%)	4 (2.45%)	
PROM			0.044
No	515 (78.39%)	116 (71.17%)	
Yes,<18 h	59 (8.98%)	14 (8.59%)	
Yes,≥18 h	83 (12.63%)	33 (20.25%) *#	
Prenatal antibiotic use			0.362
No	580 (88.28%)	148 (90.80%)	
Yes	77 (11.72%)	15 (9.20%)	
Prenatal dexamethasone use			0.003
No	506 (77.02%)	143 (87.73%)	
Yes	151 (22.98%)	20 (12.27%)	
Placenta previa			0.002
No	596 (90.72%)	160 (98.16%)	
Yes	61 (9.28%)	3 (1.84%)	
Maternal autoimmune diseases			< 0.001
No	646 (98.33%)	151 (92.64%)	
Yes	11 (1.67%)	12 (7.36%)	
Gestational diabetes			0.656
No	596 (90.72%)	146 (89.57%)	
Yes	61 (9.28%)	17 (10.43%)	
Pregnancy hypertension			0.771
No	609 (92.69%)	150 (92.02%)	
Yes	48 (7.31%)	13 (7.98%)	
ICP			0.024
No	629 (95.74%)	162 (99.39%)	
Yes	28 (4.26%)	1 (0.61%)	
Sex			0.292
Male	365 (55.56%)	98 (60.12%)	
Female	292 (44.44%)	65 (39.88%)	
Delivery mode			0.001
Vaginal delivery	145 (22.07%)	75 (46.01%)	
Operative vaginal delivery	7 (1.07%)	6 (3.68%)	
Cesarean delivery	505 (76.86%)	82 (50.31%) *#	
Apgar score			0.235
8–10	528 (80.37%)	123 (75.46%)	
4–7	111 (16.89%)	32 (19.63%)	
0–3	18 (2.74%)	8 (4.91%)	
MAS			0.002
No	651 (99.09%)	156 (95.71%)	
Yes	6 (0.91%)	7 (4.29%)	
NRDS			0.783
No	550 (83.71%)	135 (82.82%)	
Yes	107 (16.29%)	28 (17.18%)	
Wet lung of newborn			0.602
No	596 (90.72%)	150 (92.02%)	
Yes	61 (9.28%)	13 (7.98%)	

GA: Gestation age. BW: Birth weight. PROM: Premature rupture of membrane. MAS: Meconium aspiration syndrome. ICP: Intrahepatic cholestasis of pregnancy. NRDS: neonatal respiratory distress syndrome

Compared to the first factor of this classification: *, *P* < 0.05

Compared to the second factor of this classification: #, *P* < 0.05

### Univariate analysis

The results of the univariate analysis were demonstrated in Table 2. Through univariate logistic regression, we found that GA and BW were positively associated with CRP ≥ 8 mg/L, the risk of CRP ≥ 8 mg/L increased by 26% for one week increase in GA (*P* < 0.001);Antenatal steroids, placenta previa, and delivery mode(Cesarean delivery) were negatively associated with CRP ≥ 8 mg/L, with a 53%,82% and 69% reduction in the risk of CRP ≥ 8 mg/L respectively (All *P* < 0.01); PROM (≥ 18 h), maternal autoimmune diseases and MAS were positively associated with CRP ≥ 8 mg/L, with an increased risk of CRP ≥ 8 mg/L by 77%, 367% and 387%, respectively (All *P* < 0.05). (Table 2)

**Table 2:** Univariate analysis for incidence of CRP ≥ 8 mg/L [*n* = 820, Mean ± SD, *n* (%)]

	Statistics	OR (95%CI)	*P*-value
Maternal fever			
No	807 (98.41%)	1.0	
Yes	13 (1.59%)	1.81 (0.55, 5.96)	0.328
PROM			
No	631 (76.95%)	1.0	
Yes,<18 h	73 (8.90%)	1.05 (0.57, 1.95)	0.869
Yes,≥18 h	116 (14.15%)	1.77 (1.12, 2.77)	0.014
Antenatal antibiotic use			
No	728 (88.78%)	1.0	
Yes	92 (11.22%)	0.76 (0.43, 1.37)	0.363
Prenatal dexamethasone use			
No	649 (79.15%)	1.0	
Yes	171 (20.85%)	0.47 (0.28, 0.77)	0.003
Placenta previa			
No	756 (92.20%)	1.0	
Yes	64 (7.80%)	0.18 (0.06, 0.59)	0.005
Maternal autoimmune diseases			
No	797 (97.20%)	1.0	
Yes	23 (2.80%)	4.67 (2.02, 10.78)	<0.001
Gestational diabetes			
No	742 (90.49%)	1.0	
Yes	78 (9.51%)	1.14 (0.65, 2.01)	0.656
Pregnancy hypertension			
No	759 (92.56%)	1.0	
Yes	61 (7.44%)	1.10 (0.58, 2.08)	0.771
ICP			
No	791 (96.46%)	1.0	
Yes	29 (3.54%)	0.14 (0.02, 1.03)	0.053
Sex			
Male	463 (56.46%)	1.0	
Female	357 (43.54%)	0.83 (0.58, 1.18)	0.293
GA	36.07 ± 3.30	1.26 (1.18, 1.34)	<0.001
BW	2574.49 ± 814.88	1.00 (1.00, 1.00)	<0.001
Delivery mode			
Vaginal delivery	220 (26.83%)	1.0	
Operative vaginal delivery	13 (1.59%)	1.66 (0.54, 5.11)	0.379
Cesarean delivery	587 (71.59%)	0.31 (0.22, 0.45)	<0.001
Apgar score			
8–10	651 (79.39%)	1.0	
4–7	143 (17.44%)	1.24 (0.80, 1.92)	0.342
0–3	26 (3.17%)	1.91 (0.81, 4.49)	0.139
MAS			
No	807 (98.41%)	1.0	
Yes	13 (1.59%)	4.87 (1.61, 14.69)	0.005
NRDS			
No	685 (83.54%)	1.0	
Yes	135 (16.46%)	1.07 (0.68, 1.68)	0.784
Wet lung of newborn			
No	746 (90.98%)	1.0	
Yes	74 (9.02%)	0.85 (0.45, 1.58)	0.602

Result variable: CRP ≥ 8 mg/L

Exposure variable: sex, GA, BW, delivery mode, maternal fever, placenta previa, antenatal antibiotic use, PROM, pregnancy hypertension, gestational diabetes, ICP, prenatal dexamethasone use, maternal autoimmune diseases, Apgar score, RDS, MAS, wet lung of newborn

Adjust for: none

### The relationship between GA/PROM/antenatal steroids/maternal autoimmune diseases/delivery mode/MAS and incidence of CRP ≥ 8 mg/L

To further investigate the association of GA, PROM, antenatal steroids, maternal autoimmune diseases, delivery mode and MAS with CRP ≥ 8 mg/L, we used the multivariate logistic regression analysis method. By stepwise inclusion of covariates (sex, GA, BW, maternal fever, placenta previa, antenatal antibiotic use, PROM, pregnancy hypertension, gestational diabetes, ICP, MAS, antenatal steroids, maternal autoimmune diseases.), we separately established two models to illustrate the stability of these associations. Our results showed that larger GA (OR 1.13,95%CI: 1.00-1.28, *P* = 0.042), PROM(≥ 18 h) (OR 2.39,95%CI: 1.35–4.23, *P* = 0.003) and maternal autoimmune diseases (OR 5.30,95%CI: 2.15–13.07, *P* < 0.001) had a significant association with CRP ≥ 8 mg/L, and these relationships were stable even when we adjusted for confounding factors in Table 2. Indicated that GA, PROM (≥ 18 h) and maternal autoimmune diseases were independent risk factors for CRP ≥ 8 mg/L. The risk of CRP ≥ 8 mg/L increased with the increasing GA. That is for every one-week increase in the GA, there was a 13% increased risk for CRP ≥ 8 mg/L. At the same time, cesarean delivery (OR 0.40, 95%CI: 0.26–0.60, *P* < 0.001) had a significantly negative correlation with CRP ≥ 8 mg/L. (Table 3)

**Table 3:** The relationship between perinatal factors and incidence of CRP ≥ 8 mg/L in different models

	Model 1			Model 2	
	OR (95%CI)	*P*-value		OR (95%CI)	*P*-value
GA	1.16 (1.04, 1.30)	0.006		1.13 (1.00, 1.28)	0.042
PROM					
No	1.0			1.0	
Yes,<18 h	1.28 (0.67, 2.44)	0.448		1.21 (0.62, 2.38)	0.578
Yes,≥18 h	2.75 (1.66, 4.55)	< 0.001		2.39 (1.35, 4.23)	0.003
Prenatal dexamethasone use					
No	1.0			1.0	
Yes	1.56(0.83,2.94)	0.170		1.42(0.70,2.89)	0.328
Maternal autoimmune diseases					
No	1.0			1.0	
Yes	4.03 (1.70, 9.52)	0.002		5.30 (2.15, 13.07)	<0.001
Delivery mode					
Vaginal delivery	1.0			1.0	
Operative vaginal delivery	1.10 (0.34, 3.48)	0.877		1.29 (0.40, 4.22)	0.672
Cesarean delivery	0.37 (0.25, 0.54)	< 0.001		0.40 (0.26, 0.60)	<0.001
MAS					
No	1.0			1.0	
Yes	2.59(0.84,8.03)	0.099		2.89(0.90,9.26)	0.073

Result variable: CRP ≥ 8 mg/L

Exposure variable: GA, PROM, prenatal dexamethasone use, maternal autoimmune diseases, delivery mode, MAS.

Model 1 in GA adjusted for: sex, BW.

Model 2 in GA adjusted for: sex, BW, maternal fever, placenta previa, antenatal antibiotic use, PROM, pregnancy hypertension, gestational diabetes, ICP, MAS, prenatal dexamethasone use, maternal autoimmune diseases, delivery mode

Model 1 in PROM adjusted for: sex, gestational age, BW.

Model 2 in PROM adjusted for: sex, GA, BW, maternal fever, placenta previa, delivery mode, antenatal antibiotic use, pregnancy hypertension, gestational diabetes, ICP, MAS, prenatal dexamethasone use, maternal autoimmune diseases

Model 1 in prenatal dexamethasone use adjusted for: sex, gestational age, BW.

Model 2 in prenatal dexamethasone use adjusted for: sex, GA, BW, maternal fever, placenta previa, antenatal antibiotic use, PROM, pregnancy hypertension, gestational diabetes, ICP, MAS, maternal autoimmune diseases, delivery mode

Model 1 in maternal autoimmune diseases adjusted for: sex, gestational age, BW.

Model 2 in maternal autoimmune diseases adjusted for: sex, GA, BW, maternal fever, placenta previa, antenatal antibiotic use, PROM, pregnancy hypertension, gestational diabetes, ICP, MAS, prenatal dexamethasone use, delivery mode

Model 1 in delivery mode adjusted for: sex, gestational age, birth weight

Model 2 in delivery mode adjusted for: sex, GA, BW, maternal fever, placenta previa, antenatal antibiotic use, PROM, pregnancy hypertension, gestational diabetes, ICP, MAS, prenatal dexamethasone use, maternal autoimmune diseases

Model 1 in MAS adjusted for: sex, gestational age, birth weight

Model 2 in MAS adjusted for: sex, GA, BW, maternal fever, placenta previa, antenatal antibiotic use, PROM, pregnancy hypertension, gestational diabetes, ICP, prenatal dexamethasone use, maternal autoimmune diseases, delivery mode

### The non-linear relationship between GA and incidence of CRP ≥ 8 mg/L

**Fig. 1:**
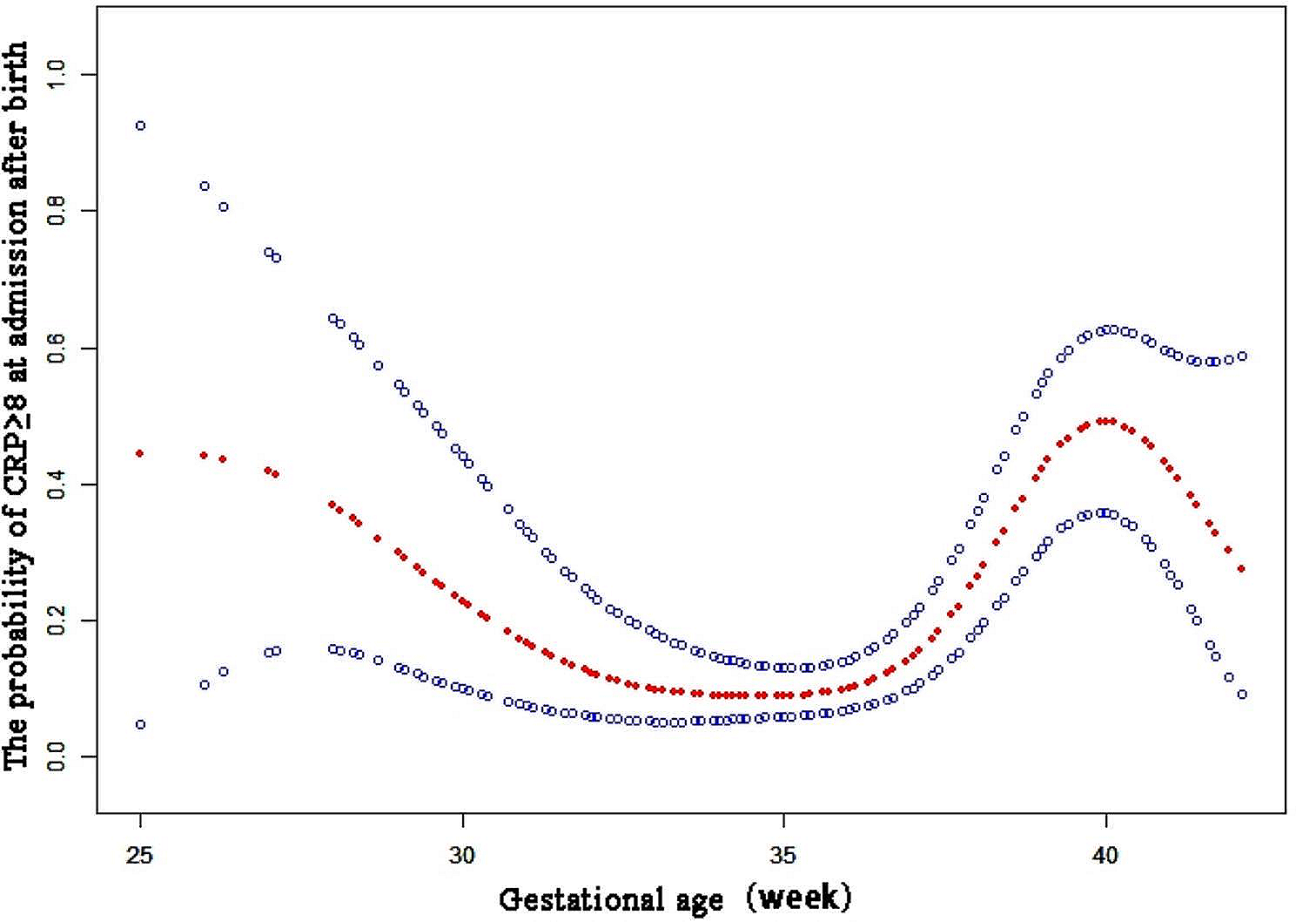
Non-linear relationship between gestational age and incidence of CRP ≥ 8 mg/L. Result variable: incidence of CRP ≥ 8 mg/L. Exposure: gestational age. Adjust for maternal fever, placenta previa, antenatal antibiotic use, PROM, pregnancy hypertension, gestational diabetes, ICP, meconium-stained amniotic fluid, prenatal dexamethasone use, maternal autoimmune diseases

In the present research, we analyzed the non-linear relationship between gestational age and incidence of CRP ≥ 8 mg/L (Fig. 1). A smooth curve and the result of generalized additive model show that the relationship between GA and incidence of CRP ≥ 8 mg/L was non-linear after adjusting for maternal fever, placenta previa, antenatal antibiotic use, PROM, pregnancy hypertension, gestational diabetes, ICP, meconium-stained amniotic fluid, antenatal steroids, maternal autoimmune diseases.

We used both logistic regression and two-piecewise logistic regression to fit the association and select the best fit model based on *P*-values for the log likelihood ratio test. Because the *P*-values for the log likelihood ratio test were less than 0.05, we chose two-piecewise logistic regression for fitting the association between gestational age and incidence of CRP ≥ 8 mg/L because it could accurately represent the relationship (Table 4). By two-piecewise logistic regression and a recursive algorithm, we calculated the inflection point to be 33.9 weeks. On the left side of the inflection point, the effect size and 95%CI were 0.72, 0.61–0.85, respectively. On the right side of the inflection point, the effect size and 95%CI were 1.61, 1.43–1.81, respectively. Which means that from 25 weeks to 33.9 weeks, the risk of incidence of CRP ≥ 8 mg/L was reduced by 28% with one week increased (*P* < 0.001), and from 33.9weeks to 42 weeks, the risk of incidence of CRP ≥ 8 mg/L was increased by 61% with one week increased. (*P* < 0.001). (Table 4)

**Table 4:** Threshold effect analysis of GA and incidence of CRP ≥ 8 mg/L using piece-wise logistic regression

	OR (95%CI)	*P*-value
Model 1		
One-line slope	1.25(1.15, 1.36)	<0.001
Model 2		
Turning point(K)	33.9	
<K slope 1	0.72(0.61, 0.85)	<0.001
>K slope 2	1.61(1.43, 1.81)	<0.001
LRT test	0.001	

Abbreviation: LRT, Likelihood Ratio Test

Result variable: incidence of CRP ≥ 8 mg/L.

Exposure variable: GA.

Adjust for maternal fever, placenta previa, antenatal antibiotic use, PROM, pregnancy hypertension, gestational diabetes, ICP, meconium-stained amniotic fluid, prenatal dexamethasone use, maternal autoimmune diseases

## Discussion

CRP status is closely related to the health status of the human body. As a non-specific marker of inflammatory response, its status changes during infections, autoimmune diseases, surgeries, cancers and cardiovascular diseases, etc. Different CRP status in the blood reflect different pathological conditions. Such changes can help physicians to enhance their judgment of the disease, which is of great significance in shortening the diagnosis time and preventing and controlling the disease progression at an early stage. We have systematically analyzed perinatal factors at birth that may affect CRP status, quantified the magnitude of the effect of different factors on CRP status, and innovatively found a nonlinear relationship between gestational age and CRP status. These will help physicians in the early judgment of EOS.

Our research found a reduced risk of CRP ≥ 8 mg/L with cesarean delivery compared to vaginal delivery. This may be due to the fact that vaginal delivery is an emergency state for neonates, which can stimulate the body to produce a large number of hormones such as glucocorticoids or catecholamine hormones, thus affecting the function of neutrophils and the production of inflammatory factors, at the same time, vaginal or forceps delivery is easy to cause certain tissue damage to neonates [20]. It was reported that the serum CRP status of healthy neonates born vaginally was significantly higher than that of neonates born by cesarean Sects. [21–23]. Therefore, the delivery mode is one of the most important factors affecting serum CRP status in the early postnatal period of neonates.

It was reported that higher CRP levels were found in patients with active serosits [24], arthritis [25], and myositis [26]. And high CRP levels were associated with high cardiometabolic risk and clinical disease activity in systemic lupus erythematosus (SLE) patients [27]. Our research found that maternal pregnancy with autoimmune disease was an independent risk factor for the increase of serum C-reactive protein within 48 h after birth, and the possible mechanism was related to maternal autoantibodies passing into the placenta and causing tissue damage in the neonates.

Gestational age is also an important factor affecting serum CRP status in the early postnatal period. It was reported that GA had a significantly positive effect on CRP, and compared with healthy term newborns, healthy preterm newborns had a lower and shorter CRP response [13–28]. Hofer et al. found that CRP levels of preterm newborns within 72 h after birth were lower than those of full-term newborns in both infected and non-infected states [29]. Our research also found that the risk of CRP ≥ 8 mg/L increased with the increasing GA. And every one-week increase in the GA, there was a 13% increased risk for CRP ≥ 8 mg/L. However gestational age had a non-linear relationship with incidence of CRP ≥ 8 mg/L, By two-piecewise Logistic regression and a recursive algorithm, our further research calculated the inflection point to be 33.9 weeks which means that from 25 weeks to 33.9 weeks, the risk of incidence of CRP ≥ 8 mg/L was reduced by 28% with one week increased (*P* < 0.001), and from 33.9 weeks to 42 weeks, the risk of incidence of CRP ≥ 8 mg/L was increased by 61% with one week increased.(*P* < 0.001).This was an interesting phenomenon never reported before to our knowledge. Preterm births were associated with multiple risk factors that are linked with increased oxidative stress [30]. Among individual biomarkers, macrophage migration inhibitory factor (MIF), interleukin-10, CRP, and tumor necrosis factor-αwere statistically significant predictors of preterm birth [31]. We reasoned the causes were that when GA was smaller than 33.9 weeks, the smaller the gestational age, the more adverse maternal stress, and the greater possibility of intrauterine infection. When GA was greater than 33.9 weeks, Dimitrios reported [32] that term newborns had a more pronounced CRP response in comparison to preterm newborns when GA was greater than 34 weeks, and the CRP values in 24 and 36 h after birth in term newborns were significantly higher than that in preterm newborns. Our results were agreeable with those reported in the literature.

It was reported that the serum CRP status of full-term neonates with MAS was significantly higher than that of the control group at the early stage after birth [29]. In our univariate analysis research, the incidence of CRP ≥ 8 mg/L in MAS was 4.87 times compared without MAS (*P* = 0.005). After adjust for sex, GA, BW, maternal fever, placenta previa, antenatal antibiotic use, PROM, pregnancy hypertension, gestational diabetes, ICP, antenatal steroids, maternal autoimmune diseases and delivery mode, the incidence of CRP ≥ 8 mg/L in MAS was 2.89 times compared without MAS (OR 2.89, 95%CI: 0.90–9.26, *P* = 0.073), which indicated MAS had a positive correlation on CRP but in this research, it may be limited by the sample size. MAS was a potential independent risk factor for CRP ≥ 8 mg/L. There are two possible mechanisms. First, meconium contamination of amniotic fluid is a marker of fetal maturity, which is commonly seen in full-term newborns, and full-term is an important factor for the increase of serum CRP in the early stage after birth [33]. On the other hand, MAS may occur when amniotic fluid is contaminated by meconium. Meconium can induce chemical inflammation in lung tissue, promote the accumulation and activation of inflammatory cells in alveolar, enhance the expression and release of pro-inflammatory cytokines, and promote the production of oxygen free radicals, resulting in tissue damage [34].

In summary, we are of the opinion that CRP status must be interpreted in the context of an newborns’ clinical condition and not used alone to guide clinical antibiotic decision making.

## Strengths and limitations

Our research has some advantages. (1) We use multiple Logistic regression to quantify the independent factor in influencing CRP; (2) we describe the association between GA and incidence of CRP ≥ 8 mg/L, solve the non-linear problem in this research and further explored this point. (3) Our results show that CRP status within 48 h after birth is affected by many non-infectious factors, and the increase of CRP is not simply caused by infection which could guide us the rational use of antibiotics in clinical practice.

There are some limitations in this research. Our research only included neonates admitted to the NICU and did not assess CRP status in healthy neonates, which may have resulted in some selection bias.

## Conclusions

GA, PROM, maternal autoimmune diseases, and cesarean delivery were all independent influences on neonatal CRP ≥ 8 mg/L on admission, and there was a nonlinear relationship between GA and neonatal CRP ≥ 8 mg/L on admission.

## Data Availability

All data generated for this analysis are available from the corresponding author upon reasonable quest from qualified researchers.
